# Community mask wearing as a COVID-19 preventive measure, its barriers, and motivators among rural households of Uganda: A mixed methods approach

**DOI:** 10.1371/journal.pgph.0000485

**Published:** 2022-07-13

**Authors:** Grace Biyinzika Lubega, Hilbert Mendoza, Juvenal Nkeramahame, Filimin Niyongabo, Joviah Gonza, Betty Nakachwa, David Musoke

**Affiliations:** 1 Department of Disease Control and Environmental Health, School of Public Health, College of Health Sciences, Makerere University, Kampala, Uganda; 2 Faculty of Medicine and Health Sciences, Department of Family Medicine and Population Health, University of Antwerp, Antwerp, Belgium; 3 FIND, The Global Alliance for Diagnostics, Geneva, Switzerland; University of Michigan, UNITED STATES

## Abstract

Adherence to mask wearing has the potential to reduce coronavirus disease 2019 acquisition risk. However, there is limited information about community mask wearing and its predictors among rural populations. This study aimed to assess the level of adherence to community mask wearing as a COVID-19 prevention measure, its barriers, and motivators among the Ugandan rural population of Wakiso District. This cross-sectional study utilised both quantitative and qualitative data collection methods. The quantitative component employed a semi-structured interviewer-administered questionnaire among 400 participants, to assess the level of adherence and associated predictors towards mask wearing. Modified Poisson regression with robust standard error estimates was used to obtain crude and adjusted prevalence ratios associated with mask wearing. Quantitative data analysis was performed using Stata 15.0 Statistical software. The qualitative component was used to further explore the barriers and motivators of community mask wearing whereseven focus group discussions among 56 community health workers were conducted. Data was analysed using a thematic approach with the help of Nvivo Version 12 software. The quantitative results showed that 70.8% (283/400) of the participants were adherent to mask wearing. Furthermore, reusable (cloth masks) were the most common form of face masks worn by the participants; 71.9% (282/400). Adequate knowledge about mask wearing as a COVID-19 prevention measure was positively associated with mask wearing (adjusted prevalence ratio (95% CI); 3.2 (1.19–8.56)). The qualitative results revealed; sensitization from health workers, provision of free masks, and fear of fines and arrests as motivators to mask wearing. Barriers to mask wearing included: inability to buy masks due to financial constraints, one-time provision of free masks, ill-fitting and worn-out masks, discomfort, and bribery. The practice of community mask wearing was sub-optimal among the study rural communities. Initiatives to scale up the practice need to be feasible for rural communities.

## Introduction

The coronavirus disease 2019 (COVID-19) pandemic has impacted the globe for two years resulting in over 4 million deaths and above 200 million confirmed cases [1]. Within the African continent, over 5 million confirmed cases and 153,778 deaths have been reported as of 19^th^ December 2021 [2]. Uganda is among the African countries greatly affected by the pandemic with 3,272 deaths as of 19^th^ December 2021 [2]. In response to the pandemic, the Ugandan government implemented non-pharmaceutical preventive measures ranging from lockdowns and personal preventive measures (Frequent hand washing with soap and water, physical distancing, and self-quarantine for those with symptoms). As of 10^th^ March 2021, Uganda embarked on COVID-19 vaccination of the public [3]. However, the country is still behind in achieving its vaccination target as approximately only 18% of her total population have either been fully or partially vaccinated as of 20^th^ December 2021 [4]. Consequently, non-pharmaceutical prevention measures are still of paramount importance in the curbing of community COVID-19 transmission.

Community mask wearing is one of such non-pharmaceutical COVID-19 prevention measures. Masks prevent the transmission of COVID-19 via two mechanisms. Firstly, masks prevent severe acute respiratory syndrome coronavirus 2 (SARS-CoV-2) infected individuals from exposing others to the virus by blocking the release of virus-containing droplets into the air. Secondly, masks protect SARS-CoV-2 uninfected wearers as they do not allow virus-containing droplets to land on the mucous membranes of both the mouth and the nose. Mounting epidemiological evidence has shown community mask wearing is 50% to 70% effective in preventing community transmission of COVID-19 [5–8]. As a result, there is need to understand the level of community mask wearing and associated factors. Rural communities of Uganda are home to 73% of the population [9]. They are largely poor with limited access to health services. These communities have lower literacy rates, limited access to electricity and smartphones, and the majority cannot afford internet services. In addition, the communities are usually hard-to-reach, have poor telecommunication network, and as such may have limited access to information on COVID-19 prevention. Another challenge faced by rural settings is limited access to safe water and soap which may reduce their adherence to COVID-19 related personal hygiene measures. High numbers of people living per household in rural communities may also reduce adherence to physical distancing.

Previous studies in Uganda assessing adherence to mask wearing have revealed community mask wearing levels at 22.22%, 70.6% and 99.3% [10–13]. However, all these studies have limited generalizability of their findings as they were carried out among urban residents and used online structured questionnaires. This consequently excludes rural dwellers thus the obtained study findings cannot be generalised to the rural population. There is limited information on community mask wearing among the rural dwellers. To the best of our knowledge, only one study has researched the use of masks among Ugandan rural populations [14]. However, this study did not employ qualitative methods to enrich the understanding of barriers and facilitators of mask wearing. Consequently, we employed a mixed methods approach to investigate the level of adherence, barriers, and motivators to mask wearing among rural residents of Wakiso district, Uganda.

## Materials and methods

### Study design and setting

This study utilised a cross-sectional study design. The study area was Wakiso district located in the central region of Uganda. A detailed profile of Wakiso district is described elsewhere [15]. Wakiso is the most populated district in Uganda with an estimated population size of 2,915,200 inhabitants [16]. The average household size is 4.7 with 47% of households residing in dwellings with only one room for sleeping, and the main occupation being subsistence farming [15]. Wakiso district was chosen because it was the second highly affected district in regards to COVID-19 cases and deaths [17], in addition to its large rural population.

### Study participants, and sampling techniques

With regards to the quantitative study component, the study units were households, and the study participants were adults (≥ 18 years old) who resided in these households. Using the Kish and Leslie formula for cross-sectional surveys [18], we calculated a sample size of minimum of 385 participants using the formula, δ) assuming a sampling error of 5%, a 95% confidence interval and a statistically conservative prevalence of 50% adherence to COVID-19 preventive measures (COVID -19 is a new disease therefore there are no previous studies carried out on this subject in Uganda). A non-response rate of 10% non-response rate was used. Then the sample size = *calculated sample size/(1−non response rate)* to give a sample size of 428. We did not adjust the sample size for a design effect in the study population section. However, from the data collection, we received 400 fully answered questionnaires, hence a sample size of 400. Multi-stage sampling was used to select respondents. We randomly selected 4 Health Sub Districts (HSDs) (Busiro South, Entebbe municipality, Busiro North, and Nasana municipality) from the 8 HSDs in Wakiso district. From each subdistrict (constituency), 2 sub-counties were then randomly selected. From each sub-county, 2 villages were randomly selected. From each chosen village, households were selected by systematic random sampling. The required number of participants per village was determined by dividing the sample size over the total number of villages. A total of 25 participants were selected per village for 16 villages. The interval for selection of the households was determined by dividing the approximate number of households in the selected village as per the list of households obtained from the Local council chairperson, over the required number of participants. The first household was randomly selected, and the interval was then taken into consideration to select the next household. Only one participant was selected per sampled household. In cases where there was more than one eligible person, simple random sampling was used to select the respondent.

For the qualitative component of the study, 7 focus group discussions (FGDs) were held among 52 community health members (CHWs) in Busiro South HSD. This HSD was chosen due to its accessibility and strong CHW presence in Wakiso district [19]. Each FGD consisted of 8 to 10 participants and lasted for approximately one and a half hours. The CHWs were purposively selected with the help of Wakiso district office. We chose to hold FGDs with CHWs because they are more knowledgeable on the health matters within their community than ordinary residents and they form the first level of the health workforce in Uganda. In addition, CHWs were among the exempted groups that were able to make home visits in their communities due to their role in the fight against COVID-19 and therefore had a better understanding on mask wearing practices within their communities.

### Data collection and data quality

Data collection was carried out between May to June 2021. For our quantitative component of the study, data was collected using a semi-structured interviewer-administered questionnaire. The questionnaire was developed after an extensive review of literature related to the use of masks during the COVID-19 pandemic [10–12]. The data collection tool comprised of questions pertaining to the socio-demographic characteristics of participants, knowledge, attitudes, and practices of mask wearing in communities. Data collection was carried out by 8 trained research assistants (RAs) using KoBoCollect mobile data collection [20].

For the qualitative component of the study, a focus group discussion (FGD) guide was used to collect data. The FGD guide was also developed after extensive review of similar studies. The FGD guide had questions related to the barriers and facilitators to the use of masks in the community. The FGDs were conducted in Luganda, the most common local language spoken in Wakiso district by 2 trained RAs. One RA took notes during the sessions while the other moderated the sessions. The discussions were audio recorded and notes were taken during the FGDs. During FGDs, COVID-19 standard operating procedures (SOPs) were observed to avoid spread of virus among participants and interviewers. All the participants received 10,000 Uganda shillings as compensation for the time taken to participate in the study. All the data collection tools were developed in English and then translated to Luganda. For consistency and accuracy, the tools were developed from two previous studies in Uganda [10–12]. Furthermore tools were validated prior data collection using face validity and content validity during the pretesting of the questionnaires, in a village (East Bujagaali) that was not part of the study.

Finally, both research components of the study were done under the supervision of the co-principal investigators.

### Study variables

Consistent mask wearing was our outcome variable of interest. The respondents self-reported if they always wore masks when they were in public spaces in the last two weeks prior to the start of the study. We adopted a guilt-free strategy as described elsewhere [21] to minimize the potential of social desirability bias that is associated with self-reported practices such as mask wearing. As a result, participants chose one response from three possible responses (“yes, always”, “yes, only when necessary”, and “no”).

The independent variables included socio-demographic characteristics (sex, age, highest level of educational attainment, number of persons living in a household, monthly income, and marital status), knowledge on mask wearing, and perceptions about mask wearing. Knowledge on mask wearing was assessed using one question that asked participants to correctly identify if mask wearing in public spaces was a COVID-19 preventive measure. As a result, knowledge was dichotomized to “no knowledge” for those who could not correctly identify mask wearing in public spaces as COVID-19 preventive measure and “had knowledge” for those that correctly identified. Perceptions about mask wearing was measured using a 5-point likert scale (strongly agree, agree, neutral, disagree, and strongly disagree). Participants were asked if they perceived mask wearing in public spaces to be an effective protective measure against COVID-19.

### Data analysis

For the quantitative data, descriptive analyses utilising frequencies and percentages were used for categorical variables. Mean (standard deviation) or median (Inter-Quartile Range/IQR) were used as descriptive statistics for numerical variables. The associations between mask wearing and independent variables were explored using modified Poisson regression with robust standard error estimates. Initially, at bivariate level, unadjusted prevalence ratios (PRs) were obtained for the association between mask wearing and each independent variable. Interactions between mask wearing and independent variables were as well examined. Afterwards, independent variables that had a threshold probability value (*p*-value) ≤ 0.2 at the bivariate level were added in the multivariable regression model. A stepwise selection method was applied until predictors in our final model had the lowest Akaike information criterion. A *p*-value of less than 0.05 was considered statistically significant. All the quantitative related data analyses were performed using Stata 15.0 statistical software (StataCorp Texas; USA).

For the qualitative component of the study, all audio recordings of the FGDs were translated, transcribed verbatim, and proofread by two of the researchers (GBL and HM). After proofreading, the transcripts were transferred to Nvivo version 12 for analysis. Data analysis was done using a deductive approach. Within each transcript, the researchers (GBL and HM) independently identified texts to code under two themes of barriers and facilitators of community mask wearing. Detailed emphasis was put on any information that consisted of the use of masks, barriers, facilitators, and any other experiences related to the use of masks. In each of the themes, sub themes were created based on the similarities of the extracts in each theme. Each of the researchers carried out coding independently and there after the researchers compared the two different sets of codes. Where coding was different, the researchers had a discussion to either harmonize the code or drop it from the analysis. The finalised findings results were then presented in form of quotations.

### Ethical approval

Ethical approval was obtained from Makerere University School of Public Health Research and Ethics Committee (Study registration number: SPH 2020–20). In addition, the study was registered with the Uganda National Council for Science and Technology (Study registration number: SS819ES). A written informal consent was obtained from the participants before they participated in the study.

#### Inclusivity in global research

Additional information regarding the ethical, cultural, and scientific considerations specific to inclusivity in global research is included in the supporting information (S1 Text).

## Results

### Background characteristics of participants

Table 1 shows the characteristics of our enrolled study participants. A total of 400 individuals were included in our analyses. More than half of the participants (68.3%) were female participants, and the average age was 43.5 years. The median number of individuals within a household was 5.

**Table 1:** Background characteristics of participants.

Characteristic	Categories	Frequency (n = 400)	Percentage (%)
Sex	Male Female	127 273	31.8 68.3
*Age	Mean (SD) ≤ 43.5 > 43.5	235 165	43.5 (15.9) 58.8 41.2
Participant is a Household head	Yes No	286 114	71.5 28.5
Highest educational level attainment	No formal education Primary Secondary Tertiary/ University	47 176 148 29	11.8 44.0 37.0 7.3
Marital status	Single Married (Cohabiting)	165 235	41.3 58.8
Number of people per household	Median (IQR) ≤ 5 people > 5 people	263 137	5 (3) 65.8 34.2
Monthly income per household	Median (IQR) ≤ 56.38$ > 56.38$	n = 370** 214 156	56.4$ (78.9$) 57.8 42.7

SD: Standard Deviation; IQR: Interquartile Range; 1$ = 3,547.94 Ugandan shillings.

*“Age was fit as a continuous variable in all models, but discretized in the table to provide a picture of the overall age distribution”.

**30 participants declined to reveal their monthly income.

### Knowledge, attitudes, and practice of mask wearing

With regards to mask wearing, the majority of participants (70.8%) reported wearing masks consistently in public spaces 2 weeks prior to the start of this study. Furthermore, reusable (cloth) masks were the most frequent (71.9%) form of facial masks possessed by the participants. Most; 96.8% and 95.8% of the participants were knowledgeable of mask wearing as a COVID-19 prevention measure and had positive attitudes towards mask wearing respectively (Table 2).

**Table 2:** Knowledge, attitudes, and practice of mask wearing.

Variable	Categories	Frequency (n = 400)	Percentage (%)
Consistent mask wearing in public spaces in the past 2 weeks	Adherent Non-adherent	283 117	70.8 29.3
Possession of a mask	Yes No	392 8	98.0 2.0
Type of facial mask	Disposable masks only Reusable (cloth) masks only Both	21 282 89	5.4 71.9 22.7
Wash reusable masks after how many days of wearing	≤ 1 day > 1 day	n = 371* 313 58	84.4 15.6
Dispose mask after how many days of wearing	≤ 1 day > 1 day	n = 110** 65 45	59.1 40.9
Knowledge of mask wearing as a preventive measure	No knowledge Had knowledge	13 387	3.3 96.8
Sources of COVID-19 preventive measures information*	Family member Health staff Phone Radio Television Church/mosque Community member Social media	58 189 84 354 253 188 203 29	14.6 47.6 21.2 89.2 63.7 47.4 51.1 7.3
Wearing a mask in public is a good protective measure against COVID-19	Disagree Neutral Agree Strongly agree	6 11 202 181	1.5 2.8 50.5 45.3

*371 is a sum of those used cloth masks only (282) and those who used both (89).

**110 is a sum of those who used disposable masks only (21) and those who used both (89).

From the FGDs, participants attributed mask wearing to COVID-19 sensitization from health workers, provision of free masks, and fear of fines and arrests. Some participants identified the use of masks to be prominent at gatherings such as functions (weddings and burials), public transportation means, and places of worship than within their local communities. Participants reported that the sensitization and information on the use of masks was possible through the use of community radios that were managed by CHWs, mass media platforms such televisions and radios that aired news on COVID-19 (Table 3).

**Table 3:** Qualitative findings pertaining to motivators of mask wearing.

Motivator Sub theme	Example quote
sensitization from health workers	“*In our village at first*, *they* [community members] *were not believing that COVID-19 is there*, *but after being sensitized*, *nowadays some have started putting on masks which was not the case at the start when they were saying that COVID-19 is not there*. *Nowadays they put on masks*, *and they do not gather many people unlike before*.*”* **FGD 5 Participant 04**
*Provision of free masks*	*“The provision of free materials from the government like free masks encouraged us to follow the guidelines like putting on masks since they were free*.” **FGD 7 Participant 07**
Fear of fines and arrests	“…*people feared* [the police] *and this helped a lot because they would say if they* [the police] *pass by here and there is nothing to show I observe COVID-19 preventive measures and they get me*, *they will punish me*, *or scare me or even embarrass me in public*.*”* **FGD 2 Participant 07**

As shown in Table 4, the participants identified several barriers to the use of masks such as the one-time provision of masks, ill-fitting and worn-out masks, and inability to buy masks due to financial constraints. Some participants reported that masks were expensive in the long run especially among households with financial constraints and families with many members eligible to wear masks. Discomforts upon wearing masks such as breathlessness and dizziness were also identified as being common among the elderly and pregnant women. Some participants also noted that traffic police officers were taking bribes instead of apprehending COVID-19 mask defaulters in public spaces.

**Table 4:** Qualitative findings pertaining to barriers of mask wearing.

Barrier Subtheme	Example of quote
One time provision of free masks	*“The government counted children aged six years and above*, *it gave them masks only once and it did not bring them back*, *in addition*, *they were few*.*”* **FGD 2 Participant 05**
ill-fitting and worn-out masks	*“The masks that were given to us by the government in our village for example*, *me I got one and when I tried it*, *it could not reach the other ear*, *it only stopped around the nose*, *it was ill fitting*.*”* **FGD 4 Participant 03**
inability to buy masks due to financial constraints	*“…as you see even if the government gave out masks*, *they did not reach everyone or household as it was previously arranged*. When it gets to the mask *issue there is someone who can’t afford to buy even a single mask because of poverty*…*”* **FGD 1 Participant 06**
Discomforts	*“…for issues of masks for elderly that when they put on masks*, *they run short of breath”* **FGD 5 Participant 03**
Bribery	*“I was in a taxi from home going to work*, *there were some people who did not have masks*, *so when the police officer asked why some did not have masks and sanitizer*, *even the taxi driver was not putting on a mask*, *do you know what the taxi conductor did*? *He folded his thumb* [nga afuunya ekikonde], *after giving him money he accepted us to go*. *It was over*, *even although we had some people in the taxi who did not have masks*.*”* **FGD 5 Participant 04**

### Predictors of mask wearing

Table 5 presents the crude and adjusted PRs for predictor variables associated with mask wearing. We observed a significant association between knowledge about mask wearing and the practice of mask wearing. After adjustment, those who knew that mask wearing was a COVID-19 preventive measure were approximately 3 times (3.2 (1.19–8.56)) more likely to wear masks in public spaces as compared to their counterparts.

**Table 5:** Predictors of mask wearing.

Characteristic	Mask wearing in public spaces		Crude PR (95% CI)	*p*-value	Adjusted PR (95% CI)	*p*-value
	Adherent (n = 283)	Non-adherent (n = 117)				
Age* ≤ 43.5 > 43.5	169 (59.7) 114 (40.3)	66 (56.4) 51 (43.6)	0.99*(0.99–1.00)	0.99		
**Possession of a mask** Yes No	281 (99.3) 2 (0.7)	111 (94.9) 6 (5.1)	1 0.32 (0.96–1.07)	0.09		
**Knowledge of mask wearing as a preventive measure** No knowledge Had knowledge	3 (1.1) 280 (98.8)	10 (8.6) 107 (91.5)	1 3.1 (1.15–8.49)	0.02	1 3.2 (1.19–8.56)	**0.02**
**Education** No formal education Primary Secondary Tertiary	31 (11.0) 133 (47.0) 101 (35.7) 18 (6.4)	16 (13.7) 43 (36.8) 47 (40.2) 11 (9.4)	1 1.15 (0.92–1.43) 1.03 (0.82–1.31) 0.94 (0.66–1.34)	0.23 0.78 0.74		
**Gender** Male Female	87 (30.7) 196 (69.3)	40 (34.2) 77 (65.8)	1 1.05 (0.91–1.21)	0.51		
**Marital status** Single Married	121 (42.8) 162 (57.2)	44 (37.6) 73 (62.4)	1 0.94 (0.83–1.07)	0.34		
**Wearing a mask in public is a good protective measure against COVID-19** Disagree Neutral Agree	2 (0.7) 3 (1.1) 278 (98.2)	4 (3.4) 8 (6.8) 105 (89.7)	1 0.82 (0.18–3.63 2.18 (0.70–6.77)	0.79 0.18		
**Number of people per household** ≤ 5 people > 5 people	188 (66.4) 95 (33.6)	75 (64.1) 42 (35.9)	1 0.97 (0.85–1.11)	0.66		

*Age was taken as a continuous variable; PR: Prevalence ratio; CI: Confidence Interval.

## Discussion

Epidemiological evidence has shown community mask wearing is effective in preventing transmission of COVID-19 [5–8]. Our quantitative results revealed a suboptimal level of community mask wearing, and knowledge of facial mask wearing as a COVID-19 prevention measure was a predictor for mask wearing. The qualitative results further identified sensitization from health workers, provision of free masks, and fear of fines and arrests as motivators for the wearing of masks. While barriers to community mask wearing included the one-time provision of masks, ill-fitting and worn-out masks, discomforts, inability to buy masks due to financial constraints, and bribery.

In our study, the level of community mask wearing among rural communities of Wakiso District was sub-optimal. Our level of mask wearing is similar to those of another study done by Mboowa et al [11]. In their study, they reported a prevalence of mask wearing of 70.3%. However, our level of mask wearing was higher than that of a study by Amodan and colleagues (33%) [10] and lower than that in a study by Ssebuufu et al (99.3) [13]. This indifference could be because Amodan and colleagues [10] study was done in the earlier phases of the pandemic before initiatives such as mass distribution of face masks by the Ugandan government [22], and the strict enforcement of mask use in public places [23–25] were introduced in Uganda. While that of Ssebuufu et al [13] did not use guilt free questions when assessing participants self-reported practices. The suboptimal level of community mask wearing highlights the need for initiatives to scale up the practice.

Our quantitative study findings revealed that knowledge of mask wearing as a COVID-19 prevention measure was positively associated with the practice of mask wearing. The qualitative findings identified sensitization on COVID-19 through health workers as a major contributor to knowledge. This may be because having knowledge about mask wearing eventually translated into practice. However, our finding should be interpreted with caution, we observed a low percentage of participants who had no knowledge on mask wearing. As a result, this may impact the accuracy of our adjusted prevalence ratios. This finding is in line with another study that showed that those who received information on face-mask use were 2.9 times more likely to use a face mask as compared to their counterparts who never received such information [19]. This implies health education initiatives promoting the wearing of masks may, in turn, increase the level of mask wearing. In rural communities, CHWs as identified from our qualitative findings are responsible for sensitizing their communities on the use of masks as mentioned in previous studies [25].

In our study, fear due to the penalties incurred for not wearing masks was a motivator for community mask wearing. It maybe because noncompliance to the COVID-19 preventive measures in Uganda has been associated with severe beatings from the police, imprisonment, and monetary penalties [23,26,27] which rural communities may not be able to pay. This finding is inconsistent with earlier studies which showed that fear of acquiring COVID-19 was a motivator for community mask wearing [11,28,29]. This inconsistency may be because previous studies were conducted earlier in 2020 when the perceived risk of contracting COVID-19 may have been higher as compared to 2021. Possible reasons for this higher perceived risk of contracting COVID-19 earlier in 2020 might have been due to lack of vaccines and inadequate knowledge on COVID-19.

The qualitative research findings showed that the provision of free masks to Ugandans by the Government of Uganda (GOU) was a motivator for community mask wearing. The GOU committed itself to providing at least one non-medical cloth mask to all Ugandans aged 6 years and above [30]. Our finding is in line with previous studies that have shown that free distribution of masks increases uptake of community mask wearing [31,32]. This implies that communities might be more willing to adhere to COVID-19 preventive measures when provided with resources such as masks that aid in the prevention of COVID-19. While the distribution of free masks was a motivator, conversely, their one-time distribution was a barrier for mask wearing. In remotest parts of the rural communities, some members did not receive the masks and therefore were not participating in mask wearing. While in other communities the masks provided were ill fitting in size and got worn out after a few washes leaving many with no masks. The one-time provision of masks could be attributed to the lack of financial capacity of the government to have a continuous distribution of masks. Another barrier to adherence to mask wearing was the lack of finances to procure masks. This is not surprising as long before COVID-19 rural communities were facing financial constraints as this population is estimated to be living below the poverty line [9]. Naziru et al [33] also identified masks as expensive for community members to afford them. Rural communities in Uganda are home to 70% of Uganda’s population who are largely poor [9]. This necessitates constant dependency on aid to support adherence to wearing of masks to COVID-19 preventive measures.

Our study found out that the use of masks was identified with discomforts such as breathlessness, dizziness which resulted into reduced use of masks. This finding is consistent with existing literature [34–36]. This finding was common among pregnant women despite being ranked as the most at risk vulnerable groups to acquire COVID-19 [37,38]. Special attention and sensitization on mask wearing should be more emphasised among vulnerable groups. We recommend that in addition to mask wearing, use of other preventive measures such as physical distancing and staying at home among the vulnerable should be more emphasized. In this study, bribery emerged as a pertinent issue for the use of masks. Similar cases of police bribery have been reported in Kenya, Zimbabwe, South Africa, and Congo during the COVID-19 pandemic [39]. Baez-Camargo, Bukuluki [40], confirms corruption in Uganda’s health system is present in all parts of the country. Previous literature, comments on how corruption is considered normal, and how people have gone ahead to manipulate such situations for their financial benefit [41,42]. We agree with [43] for anti-corruption interventions to target the causes of the causes of corruption like low salary wages for police traffic officers. This might make enforcing officers less likely to accept or ask for bribes from law defaulters.

### Study limitations

Firstly, the cross-sectional nature of our study implies we cannot imply causality in our findings. However, our study provides information on potential predictors of mask wearing. This baseline information can further be utilised by randomized controlled trials and prospective observational studies to understand the causal relationship between these potential predictors and mask wearing. Secondly, the practice of mask wearing was self-reported, consequently, there is potential for social desirability bias. However, we used “face-saving/guilt-free” questions when assessing adherence to mask wearing to minimize this bias. The concept of “face-saving/guilt-free” questions and its impact on reducing bias is described elsewhere [21]. Thirdly, the qualitative findings revealed, CHWs shared perspectives interchangeably both as CHWs and community members and as such their views in some cases where above those of an average person in the community. Despite these limitations, our study used a mixed-methods approach for triangulation of findings. The mixed methods approach also enriches our results with regards to the practice of mask wearing. Furthermore, our study population (rural residents) was an “under-studied “population in Uganda as most studies had focussed on the urban residents [10–12].

## Conclusion

The practice of mask wearing was sub-optimal in the study rural population. This highlights the need for initiatives to scale up the practice. Initiatives such as the provision of free masks, health educational messages may promote community mask wearing according to our study findings. However, there is also a need to address barriers such as the ill-fitting masks, discomforts incurred when wearing masks, and inability to buy masks due to financial constraints, and bribery which may hinder community mask wearing.

## Supporting information

S1 Data Data set used for the study. (CSV)

S1 Text PLOS’ questionnaire on inclusivity in global research. (DOCX)
